# Association of IL-9, IL-10, and IL-17 Cytokines With Hepatic Fibrosis in Human *Schistosoma mansoni* Infection

**DOI:** 10.3389/fimmu.2021.779534

**Published:** 2021-12-14

**Authors:** Karine Garcez Schuster Franco, Fabio Jorge Ramalho de Amorim, Mário Adriano Santos, Carla Virgínia Vieira Rollemberg, Fabricia Alvisi de Oliveira, Alex Vianey Callado França, Camilla Natália Oliveira Santos, Lucas Sousa Magalhães, Rodrigo Anselmo Cazzaniga, Frederico Santana de Lima, Luciana Benevides, Vanessa Carregaro, João Santana Silva, Hugo Leite de Farias Brito, Daniel Alvarenga Fernandes, Ângela Maria da Silva, Roque Pacheco de Almeida, Márcio Bezerra-Santos, Amélia Ribeiro de Jesus

**Affiliations:** ^1^ Image and Graphic Methods Unit, University Hospital, Federal Sergipe University, Aracaju, Brazil; ^2^ Health Science Graduate Program, Federal University of Sergipe, Aracaju, Brazil; ^3^ Laboratory of Molecular Biology, University Hospital, Federal Sergipe University, Aracaju, Brazil; ^4^ Department of Medicine, University Hospital, Federal University of Sergipe, Aracaju, Brazil; ^5^ Hepatology Service, University Hospital, Federal University of Sergipe, Aracaju, Brazil; ^6^ Department of Biochemistry and Immunology, Ribeirão Preto Medical School, University of São Paulo, Ribeirão Preto, Brazil; ^7^ Division of Pathology, University Hospital, Federal University of Sergipe, Aracaju, Brazil; ^8^ Infectology Service, University Hospital, Federal University of Sergipe, Sergipe, Brazil; ^9^ Immunology Institute of Investigation (III), National Institute of Science and Technology (INCT), Brazilian Research and Technology Council (CNPq), São Paulo, Brazil; ^10^ Department of Morphology, Federal University of Sergipe, São Cristóvão, Brazil

**Keywords:** schistosomiasis, IL-9, IL-10, IL-17, hepatic fibrosis

## Abstract

This is a case series study to evaluate immunological markers associated with schistosomiasis advanced fibrosis, including 69 patients from an endemic area from the State of Sergipe and from the Hepatology Service of the University Hospital in Sergipe, Brazil. Hepatic fibrosis was classified based on Niamey protocol for ultrasonography (US). Immune response to *Schistosoma mansoni* antigens was evaluated by stimulating peripheral blood mononuclear cells (PBMCs) from these patients with either adult worm (SWAP—10 μg/ml) or egg (SEA—10 μg/ml) antigens or purified protein derivative of turberculin (PPD—10 μg/ml) or phytohemagglutinin (PHA—1 μg/ml) for 72 h. The levels of IFN-γ, TNF-α, IL-5, IL-10, and IL-17 were measured in these supernatants by ELISA and IL-9 by Luminex. Single nucleotide polymorphisms in *IL-17*, *IL10*, and *CD209* genes were genotyped using TaqMan probe by qPCR. Higher levels of IL-9, IL-10, and IL-17 were found in PBMC supernatants of patients with advanced hepatic fibrosis. Direct correlations were detected between IL-9 and IL-17 levels with US spleen sizes, portal vein diameters, and periportal thickening. The *CD209* rs2287886 AG polymorphism patients produce higher IL-17 levels. Together, these data suggest a role of these cytokines in the immunopathogenesis of advanced fibrosis in human schistosomiasis.

## Introduction

Schistosomiasis is an infectious and parasitic disease which affects about 240 million people in 78 countries and around 700 million people live in areas at risk from this infection (1). In Brazil, schistosomiasis is mainly caused by *Schistosoma mansoni* (*S. mansoni*). Most infected individuals develop mild gastrointestinal disease, but 5% to 10% develop hepatosplenic schistosomiasis, characterized by severe liver fibrosis, splenomegaly, and portal hypertension (2). About 22 million people are currently suffering from complications of chronic *S. mansoni* infection and up to 42% of infected people have been diagnosed with periportal fibrosis. About 0.2 million deaths are attributed to chronic schistosomiasis every year (3). Severe liver fibrosis and portal hypertension hold the main responsibility for morbidity and mortality related to schistosomiasis and high cost for the healthcare and control programs of countries, placing this disease as an important public health concern (4, 5).

The mechanisms related to the development of severe forms of the disease in humans have not been clearly defined yet (6). Schistosomiasis morbidity has varied directly with egg count, but the nature of the immune response of the host is what shapes the magnitude of the immunopathology in each case (6). Immune response mediated by CD4+ T cells from the host to *S. mansoni* eggs laid in the liver and intestine, with granuloma formation, leads to fibrosis, with consequent portal hypertension (7). Studies in both, experimental models and in human, show that the initial events of granuloma formation are associated with a Th2 response (8). Additionally, in an experimental model with an advanced fibrosis resembling advanced hepatic fibrosis from human schistosomiasis, a role of Th17 response is demonstrated, whereas in human schistosomiasis, there is no clear understanding of what occurs in the later stages of fibrosis (9). Furthermore, the genetic background of the host might be involved in the modulation of immune response and pathogenesis development, as seen in many infectious diseases (10–15). Thus, functional single nucleotide polymorphisms (SNPs) may be important as they may influence the gene expression or the structure of the corresponding protein (16). The understanding of the molecular mechanisms involved in the immunopathogenesis of this disease can have important implications for intervention strategies, mainly in immunoprophylaxis, not only in schistosomiasis but also in other parasitic diseases (5, 17).

he present study evaluates the cytokine profiles of Th1 (IFN-γ, TNF-α), Th2/Treg (IL-5, IL-10), Th9 (IL-9), and Th17 (IL-17) in peripheral blood mononuclear cell (PBMC) supernatants stimulated with soluble egg (SEA) and adult worm (SWAP) antigens from patients with schistosomiasis with different stages of hepatic fibrosis and investigates whether SNPs in *IL17A*, *IL10*, and *CD209* receptor [dendritic cell-specific ICAM3-grabbing non-integrin (DC-SIGN)] are associated with this pathogenesis and influence the expression of IL-10 and IL-17 in this population.

## Materials and Methods

### Ethics Statement

This study was carried out in accordance with the recommendations of the Declaration of Helsinki. All donors or their legal guardians from whom information and biologic samples were obtained or clinical procedures were performed gave written informed consent for their participation in the study. The study was approved by the Ethics and Research Committee from the University Hospital of the Federal University of Sergipe, Brazil (CAAE 0022.0.107.000-08).

### Study Design and Patient Selection

A case series analytical study was performed in patients with schistosomiasis, comparing patients classified according to the degree of fibrosis by ultrasonography (US). A total of 69 patients were included. Most patients were recruited from the city of Ilha das Flores, an important endemic area for schistosomiasis, located in the north of the State of Sergipe, Brazil, on the banks of the São Francisco River. According to the 2007 Schistosomiasis Control Program data, there is a high frequency (46.45%) in the positivity of parasitological stool test in this municipality, provided by constant and obligatory contacts with natural sources of water, which are abundant in the municipality, whether in work activities or in daily household chores. As only a mild hepatosplenic form was present in about 2% to 7% of infected patients from this endemic area (18), patients treated at the Hepatology Service of the University Hospital (UH) at the Federal University of Sergipe were recruited. These patients were from other endemic areas of the state or even from neighboring states, such as Alagoas and Bahia, and presented severe hepatosplenic schistosomiasis, but did not have any other associated diseases. Therefore, it was possible to analyze the immune response associated to more advanced stages of the disease.

The inclusion criteria for the study were as follows: positive parasitological diagnosis based on the Kato-Katz method for *S. mansoni*, or previous treatment background, classified using abdominal ultrasonography with different degrees of fibrosis compatible with *S. mansoni* etiology. Exclusion criteria were age <5 and >70 years old, pregnant or people with diseases which affect the immune response (HIV, HTLV-1, and diabetes mellitus), people with other liver diseases associated with portal hypertension (such as hepatitis A, B, or C; cirrhosis; Budd–Chiari; portal vein thrombosis) and acute viral or bacterial infections in the moment of blood collection, and patients who have not completed the immunological evaluation. After applying the exclusion criteria, the selected patients were divided into two groups: no fibrosis (*n* = 53), all of them from the endemic area of Ilha das Flores, and with advanced fibrosis (*n* = 16), all of them from the Hepatology Service of the University Hospital (UH). The small sample size in the advanced fibrosis group is due to the rigidus selection criteria, which include only patients with confirmed hepatic fibrosis and portal hypertension, also confirmed by esophageal varices in the endoscopy, and exclude patients with comorbidities and coinfections that are very frequent in these group of patients. The small sample might reduce the power and interfere in the negative results, but the significant data are still valid.

After all, a genetic polymorphism study was performed in both groups. Unfortunately, due to problems of DNA recovery from the blood samples and low quantification of DNA, only a total of 37 of these individuals were enrolled in the SNP analysis: 28 patients from the no fibrosis group and 9 patients from the fibrosis group.

### Ultrasonography

Ultrasound exams (US) were done following the method defined by the World Health Organization at Niamey meeting, 2000, for the classification of hepatic fibrosis. The US was performed by the same sonographer on a high-resolution device, Medison brand, model: Sonoace 8000 SE. The Niamey classification consists of three stages, which result in three scores that assess intraparenchymal (IP), periportal (PT), and portal hypertension (PH) aspects. The assessment of periportal fibrosis in this protocol is analyzed by two distinct methods: a qualitative one, which considers the ultrasound aspect of the liver, evaluated by characteristic patterns, the IP score, associated with a quantitative method, which results from the measurement of two or three second-order portal branches, the PT score. In addition to these two criteria, the presence of portal hypertension (PH score) is also analyzed, which is given by the analysis of indicators of its existence, as the measure of the portal vein diameter, the presence of ascites, and the presence of collateral circulation. These scores (IP, PT, and PH) are interpreted using a table that classifies the periportal fibrosis as follows: group 1 (no sign of periportal fibrosis), group 2 (incipient periportal fibrosis not excluded), group 3 (possible periportal fibrosis), group 4 (probable periportal fibrosis), group 5 (periportal fibrosis), group 6 (advanced periportal fibrosis), and group 7 (advanced periportal fibrosis + portal hypertension). This method has been used in several previous studies (19–23).

### Immunological Evaluation

The specific antigens of *S. mansoni* used were soluble extracts of adult worm (SWAP) and soluble egg antigen (SEA) which were kindly provided by Evan Secor from the Division of Parasitic Diseases and Malaria, Centers for Disease Control and Prevention, Atlanta, Georgia. For the isolation of PBMC from heparinized blood, it was subjected to a density gradient using a Ficoll Hypaque gradient (Ficoll-Paque^™^ PLUS; GE Healthcare, USA), as previously described (24, 25). In summary, 3 × 10^6^ cells in 1 ml of RPMI 1640 (Gibco™, Thermo Fisher Scientific, Brazil) supplemented with 10% AB Rh-positive serum were either not stimulated or stimulated with specific *S. mansoni* antigens. The antigen concentration of 10 μg/ml for both SEA and SWAP was chosen because it was able to induce a response in PBMC from patients with schistosomiasis, but not from healthy controls from the same endemic area. The mitogen phytohemagglutinin (PHA) at 1 μg/ml and the purified protein derivative of turberculin (PPD) at 10 μg/ml were used as positive controls. After a 72-h incubation at 37°C in 5% CO_2_, the plates were centrifuged and the supernatants collected, aliquoted, and stored at −80°C for later cytokine dosage.

### Determination of Cytokine Levels

The levels of IFN-γ and TNF-α (Th1 profile), IL-5 and IL-10 (Th2/Treg profiles), and IL-17 (Th17 profile) cytokines were measured in these supernatants by the ELISA technique using commercially available kits (DuoSet, R&D Systems Inc., USA) on microtiter plates (Kartell SPA, Italy). The techniques described by the manufacturers were followed, except that the diluent for establishing the standard curves was the same (RPMI + 10% AB-positive sera) as for the tested samples. IL-9 (Th9) levels were measured by Luminex (Milliplex kit, Panomics Inc., Affymetrix, USA). IL-9 was only analyzed in SEA-stimulated supernatants because of the limitation of the number of samples in the kit available. The measurement techniques were also described in previous publications of our group (5, 26).The results were expressed in pg/ml based on a standard curve using the recombinant cytokines. It was not possible to measure all cytokines in all patients because we run out of some of the PBMC supernatants.

### SNP Genotyping

SNPs in *IL17A*, *IL10*, and *CD209* (DC-SIGN) gene were genotyped by allelic discriminations. In brief, genomic DNA was extracted using PureLink^®^ Genomic DNA Mini Kit (Invitrogen™, USA). The concentration and purity of DNA were quantified using NanoDrop™ (Thermo Scientific™, USA). We genotyped DNA samples using *IL17A* rs2275913, *IL10* rs1800871 and rs1800872, and *CD209* rs2287886 and rs4804803 TaqMan^®^ probe by qPCR using 7500 Real-Time PCR (Applied Biosystems, USA) following the instructions of the manufacturer. The results were assessed using TaqMan^®^ Genotyper software version 1.6.0 (Applied Biosystems, USA). Information about the analyzed SNPs are presented in **Supplementary Table 1**.

### Statistical Analyses

D’Agostino & Pearson (D’Agostino & Pearson omnibus normality test) and Shapiro–Wilk normality tests were applied to the numerical data of the groups. Differences between cytokine levels in PBMC supernatants were compared between groups with different degrees of fibrosis using the Kruskal–Wallis non-parametric test, with Dunn’s post-test. Correlations were also made between numerical variables such as cytokine levels with measurements of periportal thickening, portal vein diameter, and spleen size by Spearman’s correlation test. Allelic and genotype frequency associations in the SNPs were analyzed by Fisher exact test or Chi-square test. Odds ratio value was used to compare the groups. These analyses were performed using the GraphPad Prism program, version 6.0. A value of *α* ≤5% (*p* ≤ 0.05) was considered for statistical significance. The Hardy–Weinberg equilibrium test was performed by GENEPOP online 4.2 available at http://genepop.curtin.edu.au/.

## Results

### Demographic and Clinical Aspects of Individuals From the Endemic Area and From the University Hospital

The demographic and clinical data of patients recruited from the endemic area and from the UH, classified according to their clinical forms, are shown in **Table 1**.

**Table 1:** Demographic, clinical, and ultrasound data of individuals from the endemic area and hepatosplenic ones from the University Hospital (UH) classified according to their clinical forms.

Variables	I + HI	HE (endemic area)	HE (UH)	Statistical analyses^a^
Number	46	7	16	
Age (years)				
Range	5–73	16–45	23–76	
Mean ± SD	26 ± 14.7	28 ± 10.1	53 ± 17.1	
Median	21.5^b^	29.0	57.1^b^	*p* < 0.0001^b^
Male gender	28 (60.9%)	5 (71.4%)	9 (56.3%)	
Clinical examination				
Liver size (xiphoid process in cm)	2 ± 2.0^c^	5.7 ± 1.1^c^	3.9 ± 2.8	*p* = 0.0004^c^
Spleen size (cm)	0.96 ± 0.2^d,e^	2.7 ± 0.49^d^	2.7 ± 0.70^e^	*p* < 0.0001^d,e^
Ultrasonography				
Left liver lobe size (cm)	84.7 ± 17.48	89.8 ± 16	79.3 ± 18,41	*p* = 0.35
Spleen size (cm)	106 ± 21.2^f^	115.5 ± 21.24^g^	180.5 ± 39.07^f,g^	*p* < 0.0001^f,g^
Periportal space (mm)	2 ± 0.4^h^	1.9 ± 0.32^i^	5.6 ± 3.61^h,i^	*p* < 0.0001^h,i^
Portal vein diameter (mm)	9 ± 1.5^j^	9.9 ± 1.57	12.9 ± 2.50^j^	*p* < 0.0001^j^
Ascites	0	0	2 (12.5%)	
Niamey classification	No fibrosis (Niamey 1)	No fibrosis (Niamey 1)	Advanced fibrosis (Niamey 7)	
Esophageal varices in digestive endoscopy	Not performed	Not performed	16 (100%)	

^a–j^Kruskal–Wallis with Dunn’s post-test. I, intestinal patients; HI, hepatointestinal patients; HE, hepatoesplenic patients, UH University hospital.

It is observed that hepatosplenic patients (HE) from the UH are significantly older than the ones with intestinal (I) and hepatointestinal (HI) forms from the endemic area. All patients from the Hepatology Service of UH (*n* = 16) had advanced fibrosis on ultrasound, associated with portal hypertension (Niamey 7), mainly verified by the measurements of the spleen size, the portal vein diameter, and the periportal space and showed portal hypertension signs, confirmed by detection of esophageal varices in upper digestive endoscopy. Patients from the endemic area (*n* = 53) did not have advanced fibrosis, being classified as Niamey 1.

### Cellular Immune Response in Individuals Classified According to the Degrees of Fibrosis Determined by Ultrasound

**Figure 1:**
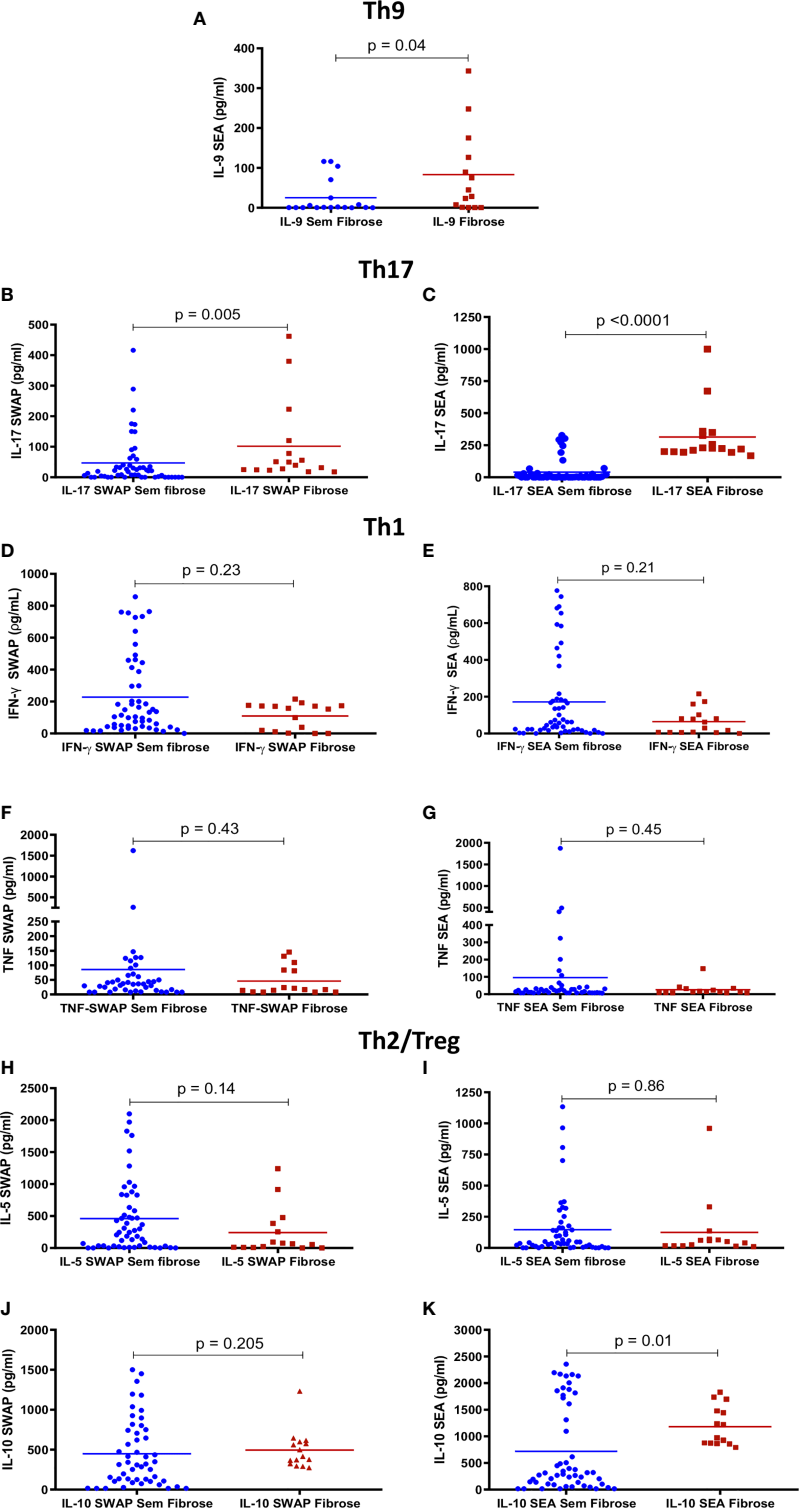
Peripheral blood mononuclear cell (PBMC)-specific cytokines response to *Schistosoma mansoni* antigens in patients with different degrees of fibrosis. Comparisons between the levels of IL-9 **(A)**, IL-17 **(B, C)**, IFN-γ **(D, E)**, TNF-α **(F, G)**, IL-5 **(H, I)**, and IL-10 **(J, K)** in supernatants from PBMC cultures stimulated with adult worm (SWAP) and egg (SEA) antigens in individuals with different degrees of fibrosis by the Niamey criteria (no fibrosis and advanced fibrosis). IL-9 was only analyzed in SEA-stimulated supernatants because of the limitation of the number of samples in the kit available. The data represent individual values and the averages are shown. Comparisons were analyzed using the Mann–Whitney *U* test.

The levels of cytokines IFN-γ, TNF-α, IL-5, IL-9, IL-10, and IL-17 were compared in the groups of patients without fibrosis (classified as Niamey 1) (*n* = 53) and with advanced fibrosis (classified as Niamey 7) (*n* = 16) (**Figure 1**). IL-17 levels were significantly higher in PBMC supernatants stimulated with SWAP and SEA in patients with fibrosis (**Figures 1B, C**). IL-9 and IL-10 levels were significantly higher in the PBMC supernatants stimulated with SEA in patients with fibrosis (**Figures 1A, K**). There were no significant differences in the levels of IFN-γ, TNF-α, and IL-5 in the PBMC supernatants stimulated with SEA and SWAP (**Figures 1D–I**). All patients presented a response to PPD and PHA stimulations.

### Correlations Between Cellular Immune Response and Ultrasound Data Associated With Fibrosis and Portal Hypertension

**Figure 2:**
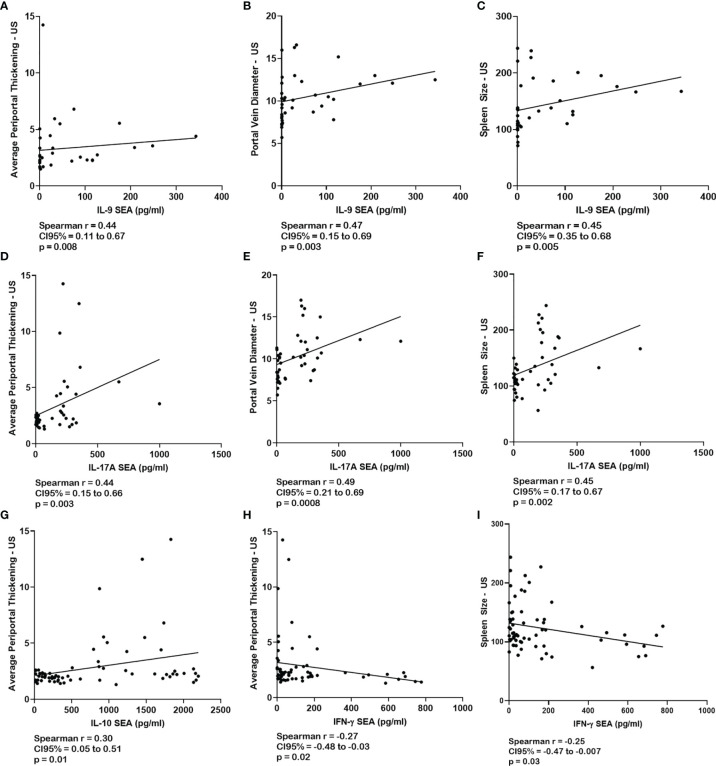
Correlations between the response of peripheral blood mononuclear cell (PBMC)-specific cytokines to *S. mansoni* antigens and the ultrasound data associated with fibrosis and portal hypertension. Correlations between the levels of IL-9 **(A**–**C)**, IL-17 **(D**–**F)**, IL-10 **(G)**, and IFN-γ **(H, I)** in the supernatants from PBMC cultures stimulated with soluble egg antigen (SEA) and the periportal thickening, portal vein diameter, and the spleen size measured on ultrasound. Correlations were analyzed using Spearman’s correlation test.

Correlations were made between the levels of IFN-γ, TNF-α, IL-5, IL-9, IL-10, and IL-17 cytokines and the average of the measurements of the periportal space, the portal vein diameter, and the spleen, taken by ultrasound (**Figure 2**). There was a direct correlation between these measurements and the levels of IL-9 and IL-17 cytokines in response to SEA (**Figures 2A–F**). There was also a direct correlation between IL-10 levels in response to SEA and the average measurements of periportal space (**Figure 2G**). IFN-γ in response to SEA showed an inverse correlation with periportal thickening and spleen size (**Figures 2H, I**). There were no significant correlations between these measurements and the levels of TNF-α and IL-5 (**Supplementary Table 2**).

### Genotyping of *IL17A*, *IL10*, and *CD209* SNPs

The SNPs in *IL17A* (rs2275913), *IL10* (rs1800871 and rs1800872), and *CD209* (rs2287886 and rs4804803) genes were genotyped in the no fibrosis patients (classified as Niamey 1) (*n* = 28) and fibrosis patients (classified as Niamey 7) (*n* = 9). No deviation was found in the Hardy–Weinberg equilibrium test. There were no significant differences in genotype and allelic comparisons between schistosomiasis patients with fibrosis and without fibrosis, despite the differences in genotype frequency distributions of *IL-10* (rs1800871 and rs1800872) and *CD209* (rs2287886 and rs4804803) SNPs (**Supplementary Table 3**).

### *CD209* rs2287886 SNP Is Associated With Differences in IL-17 Levels in Schistosomiasis Patients

When we compared the amount of IL-17 quantified in the PBMC supernatants of all patients included in the genetic analysis together, stratified according to genotypes, we found an association between higher levels of IL-17 in the AG genotype to *CD209* rs2287886 when compared with the GG genotype in non-stimulated PBMC supernatants (*p* = 0.051) but not in the SWAP (*p* = 0.063) nor SEA (*p* = 0.964)-stimulated PBMC supernatants (Mann–Whitney test—**Supplementary Figure 1**). There were no other differences between the genotype of other SNPs assessed and the cytokine measurement found in the PBMC supernatants in this population when all patients included in the genetic analysis were evaluated together.

Complementary to this, we analyzed the levels of IL-17 and IL-10 in non-stimulated and stimulated with SWAP or SEA PBMC supernatants according to different genotypes for the SNPs studied in each group of patients separately (no fibrosis and advanced fibrosis) (**Supplementary Figure 2**). We observed significant differences between cytokine dosages when advanced fibrosis and no fibrosis patients with the same genotype for the SNPs were compared (**Supplementary Figure 2**).

## Discussion

The present study detects a predominance of IL-17 (Th17) and IL-9 (Th9) production in hepatosplenic patients with advanced fibrosis in PBMC cultures stimulated with *S. mansoni* antigens. In addition, there is a direct correlation between these cytokine levels and data related to the severity of fibrosis, which suggests an important association of the Th17 and Th9 response with the severe form of human schistosomiasis. Besides, the study demonstrates a predominance of IL-10 production in hepatosplenic patients with advanced fibrosis in cultures stimulated with SEA and a correlation between this response and the average measurements of periportal space, indicating a possible Th2/Treg response remaining from the initial periportal fibrosis or an attempt to downmodulate the fibrosis.

Many studies show the importance of the immune response to parasite antigens in the pathogenesis of schistosomiasis. Studies in experimental models have shown that cytokines are important factors in the formation and modulation of the immune response to *S. mansoni* egg. Most experimental studies demonstrate the role of Th2 cytokines (IL-4, IL-5, and IL-13) in the formation of granuloma (27–29) and of the Th17 response in advanced fibrosis (9, 30). In human schistosomiasis, the influence of the Th2 response and the TGF-β cytokine in periportal fibrosis has been demonstrated (5), but they have failed to identify the immune response profile associated with advanced fibrosis, because the studied patients did not present advanced fibrosis. To our knowledge, this is the first study that demonstrates the association of these cytokines with advanced fibrosis in human schistosomiasis.

The present study observes a predominance of IL-17 (Th17) production in hepatosplenic patients with advanced fibrosis in cultures stimulated with *S. mansoni* antigens. In addition, there is a direct correlation between these cytokine levels with the spleen size, portal vein diameter, and periportal thickening, data related to the severity of the disease, which suggests an important association of the Th17 response with the pathogenesis of the severe form of human schistosomiasis. These findings are in accordance with literature data in an experimental model that develops severe fibrosis, corresponding to advanced human fibrosis, known as Symmers’ fibrosis. In these mice, the IL-23 and the induction of IL-17 have been shown to play a critical role in the immunopathology of schistosomiasis (31). Kalantari et al. (32) demonstrated that CD209a-deficient CBA mice display decreased Th17 responses and are protected from severe immunopathology. A recent study found that acute schistosome infection induced a transient Th17 response to cathepsin B1 cysteine proteases secreted by the worms and that this early Th17 response may determine the pathogenic progression of the infection (7, 33). The Th17 response has also been associated with the pathogenesis of other human helminth infections (34).

The present study also demonstrates a predominance of IL-9 production in hepatosplenic patients with advanced fibrosis in SEA-stimulated cultures, in addition to correlations between this response and the spleen size, portal vein diameter, and periportal thickening, thus suggesting a role of the Th9 response in the pathogenesis of the severe form of human schistosomiasis. In mice infected with *S. japonicum*, proliferation of Th9 cells and elevated levels of IL-9 have been demonstrated. In addition, dynamic changes of Th9 and IL-9 were synchronous with the developmental trend of hepatic egg granulomatous inflammation, suggesting that Th9 cells might be a new subset in the pathogenesis of schistosomiasis (35). Several studies demonstrate the role of this cytokine in several classes of inflammatory diseases. Treatment with IL-9 neutralizing antibodies effectively attenuates liver inflammation and necrosis (36). In allergic airway disease, allergic patients have more circulating Th9 cells than non-allergic control subjects (37). Neutralization of IL-9 was able to reduce allergen-induced inflammation (38).

The only study carried out in humans by Barreto et al. (39), evaluating the association of IL-17 and IL-9 in sera from patients with several clinical forms of schistosomiasis, did not show significant differences in the levels of these cytokines in patients with different degrees of fibrosis. However, in this study, the cytokines were measured in serum samples of these patients, and they did not evaluate the specific immune response to *S. mansoni* antigens. More importantly, it is noteworthy that the study only included two patients with advanced fibrosis.

The present study shows that there is a difference in the IL-10 (Th2/Treg) levels in patients with advanced fibrosis in the supernatants of PBMC cultures when stimulated with SEA. There are no differences in the response to the SWAP, demonstrating that this response is specific to the egg antigen. Previous studies in experimental models demonstrate the IL-10 induction mediated by carbohydrates of the *Schistosoma* eggshell (40, 41). We also observed a direct correlation between these cytokine levels and periportal thickening, with no correlations between IL-10 and the US data most associated with portal hypertension (portal vein diameter and spleen size). IL-10 is known for its regulatory function, and some experimental studies show an association between its absence and accelerated liver fibrosis in schistosomiasis (42). A study in mice showed that IL-10 is an important immunoregulatory cytokine in acute schistosomiasis, but it plays no essential role in the process of immune downmodulation in chronic *S. mansoni* infection in mice (43). Jesus et al. (5) also observed an increase in IL-10 in patients with degree III hepatic fibrosis, representing the initial stages of periportal fibrosis, but in that study, there were no patients with advanced fibrosis. Our hypothesis is that the Th2/Treg response shown to occur in the early stages of liver fibrosis and reported in experimental models (44, 45) and the high IL-10 levels found in the present study in patients with advanced fibrosis represent an attempt to modulate the injury-inducing immune response but are insufficient to prevent the progression of fibrosis.

Considering previous reports of functional SNPs in these cytokine genes, in this study, the *IL17A* rs2275913, *IL10* rs1800871, *IL10* rs1800872, *CD209* rs2287886, and *CD209* rs4804803 were genotyped. Although no statistical differences were in the allelic and genotype frequencies comparisons, probably due to the small sample size, differences in the genotype frequency distributions of *IL-10* (rs1800871 and rs1800872) and *CD209* (rs2287886 and rs4804803) SNPs were observed between schistosomiasis patients with fibrosis and without fibrosis.

Here, we found an association between the presence of genotype AG for the *CD209* rs2287886 with higher levels of IL-17 in non-stimulated PBMC supernatant when compared with the GG genotype in schistosomiasis patients.

The CD209 receptor, also known by DC-SIGN, is part of a human C-type lectin receptor family and has as primary function in the recognition of conserved carbohydrate domains that are present in many pathogens, including *S. mansoni* (46). Its recognition leads DCs to produce IL-1β and IL-23 and induces Th17 responses (9). Experimental studies demonstrated that, despite similar infection protocols, there is a variation in disease severity among different mouse strains regarding schistosomiasis. While CBA mice develop large egg granulomas and severe disease due to CD209a activation to produce IL-1β and IL-23 and to induce Th17 cells, BL/6 mice develop smaller lesions, explained by a disability to induce Th17 response by the CD209a recognition pathway (47, 48).

The A allele in human *CD209* rs2287886 is associated with a higher expression of CD209 in DC from healthy donors, when compared with the G allele. Interestingly, the CDs that expressed significantly higher levels of CD209 were also more susceptible to cytomegalovirus infection (13). No patient from the fibrosis group presented the AA genotype, but a higher frequency of the AG genotype was observed in this group. Our findings that the genotype AG is associated with higher levels of IL-17 agree with a possible explanation derived from previous studies in a murine model, since the increased expression of CD209 receptor in CDs is associated with induction of Th17 response (9, 47, 48).

The lack of associations between the *IL17A* rs2275913, *IL10* rs1800871, *IL10* rs1800872, *CD209* rs2287886, and *CD209* rs4804803 alleles or genotype frequencies with the schistosomiasis clinical manifestations in this population could be due to small sample size, and further studies are necessary to rule out the influence of these SNPs in schistosomiasis pathogenesis. In spite of this, we could observe significant differences between cytokine dosages when advanced fibrosis and no fibrosis patients with the same genotype for the SNPs were compared, suggesting that this modulation may occur as a consequence of disease severity variations and not due to genetic background of the SNPs assessed.

There is no association between the levels of IL-5, TNF-α, and INF-γ and the advanced degree of fibrosis in the present study. Most studies in mice clearly show the role of Th2-type cytokines such as IL-4 (27) and IL-13 (29) in determining granulomatous lesion and hepatic fibrosis in schistosomiasis, with IL-5 being important for eosinophilia present in these granulomas (49). Shainheit et al. (50) evidenced that there was no fibrosis progression after blocking the production of INF-γ in mice. However, these mice do not have the classic Symmers’ fibrosis and portal hypertension from the schistosomiasis observed in the human species. In another mouse model, which presents fibrosis more like that which occurs in humans, it was evidenced that cytokines such as TNF-α (51) and IL-17 (31, 52) have an important role in this fibrosis process. In the human species, it has been observed that, similar to mice, the immune response of the Th2 profile (IL-5, IL-13) in response to egg antigen (SEA) is associated with the early stages of fibrosis, using the classification of Abdel-Wahab (5). However, in this study, there were no patients with more advanced hepatosplenic form. A study performed in Africa evidenced higher levels of TNF-α and IFN-γ and lower levels of IL-5 in PBMC supernatants stimulated with *S. mansoni* egg antigen in hepatosplenic individuals when compared with non-hepatosplenic ones (53). However, the patients in this study were from a malaria-endemic area and were classified only by clinical examination, and the hepatosplenomegaly may also be due to malaria. Malaria infection, in turn, could also justify the high levels of TNF-α and IFN-γ found in these patients (54).

In conclusion, the severity of fibrosis observed by ultrasonography in human schistosomiasis is associated with a Th9 and Th17 response specific to *S. mansoni* antigens. The direct correlation between the levels of IL-9 and IL-17 cytokines and the spleen size, portal vein diameter, and thickening of the periportal space reinforces the association of these cytokines with the immunopathogenesis of human schistosomiasis. The observation that IL-10 is also increased in patients with advanced fibrosis and the correlation found between IL-10 levels only with thickening of the periportal space suggest the hypothesis of an attempt of fibrosis modulation by this cytokine.

## Data Availability Statement

The raw data supporting the conclusions of this article will be made available by the authors, under a reasonable request.

## Ethics Statement

The studies involving human participants were reviewed and approved by the Ethics and Research Committee from the University Hospital of the Federal University of Sergipe, Brazil. Written informed consent to participate in this study was provided by the legal guardian/next of kin of the participants.

## Author Contributions

AJ, AS, MS, CR, and FA recruited and clinically characterized the schistosomiasis patients from the endemic area of Ilha das Flores. AF, AS, and FL recruited and clinically characterized the patients from the University Hospital of Federal University of Sergipe. KF and DF performed the ultrasound exams and analyzed and interpreted the data related to the classification of hepatic fibrosis. FA, CR, FO, CS, and LM performed the immunological evaluation experiments. CS and RC performed the SNP genotyping experiments and analyzed and interpreted the data. AJ and RA conceived and designed the study. AJ, MS, and VC supervised the research and interpreted the data. KF, HB, LB, and AJ drafted the manuscript. AJ, RA, and JS revised the manuscript. All the authors discussed the results and commented on the manuscript. All authors contributed to the article and approved the submitted version.

## Funding

This work was supported by Edital MS/CNPq/FAPITEC/SE/SES N° 06/2007 – PPSUS (Grant number 19.203-00775/2007-3), CAPES-Basic Parasitology (032/2010), and EDITAL CAPES/FAPITEC N° 11/2016 – PROEF (Grant number 88881.157436/2017-01). AJ is a scientist supported by the Brazilian Research and Technology Council (CNPq).

## Conflict of Interest

The authors declare that the research was conducted in the absence of any commercial or financial relationships that could be construed as a potential conflict of interest.

## Publisher’s Note

All claims expressed in this article are solely those of the authors and do not necessarily represent those of their affiliated organizations, or those of the publisher, the editors and the reviewers. Any product that may be evaluated in this article, or claim that may be made by its manufacturer, is not guaranteed or endorsed by the publisher.

## Supplementary Material

Supplementary Figure 1 CD209 rs2287886 SNP is associated with differences IL-17 in schistosomiasis patients. Levels of IL-17 in 37 schistosomiasis patients stratified by CD209 rs2287886 genotype. **p* < 0.05 (Mann-Whitney test).

Supplementary Figure 2 Cytokine levels in no fibrosis and advanced fibrosis schistosomiasis patients by different genotypes to IL17A rs2275913, CD209 rs2287886, CD209 rs4804803 and IL10 rs1800871 SNP. Comparisons between the levels of IL-17 and IL-10 cytokines in supernatants from PBMC cultures not stimulated (Medium) or stimulated with adult worm (SWAP) and egg (SEA) *S. mansoni* antigens from schistosomiasis patient stratified by groups of advanced fibrosis (*n*=9) and no fibrosis (*n*=28) and by genotypes to CD209 rs2287886 **(A)**, CD209 rs4804803 **(B)**, IL-17A rs2275913 **(C)**, and IL-10 rs1800871**(D)**. **p* < 0.05 (Mann-Whitney test).
