# Supplementary figures and images for: Association of IL-9, IL-10, and IL-17 Cytokines With Hepatic Fibrosis in Human Schistosoma mansoni Infection
Source: Front Immunol. 2021 Dec 14;12:779534. doi: 10.3389/fimmu.2021.779534 (PMC8712476; doi:10.3389/fimmu.2021.779534)

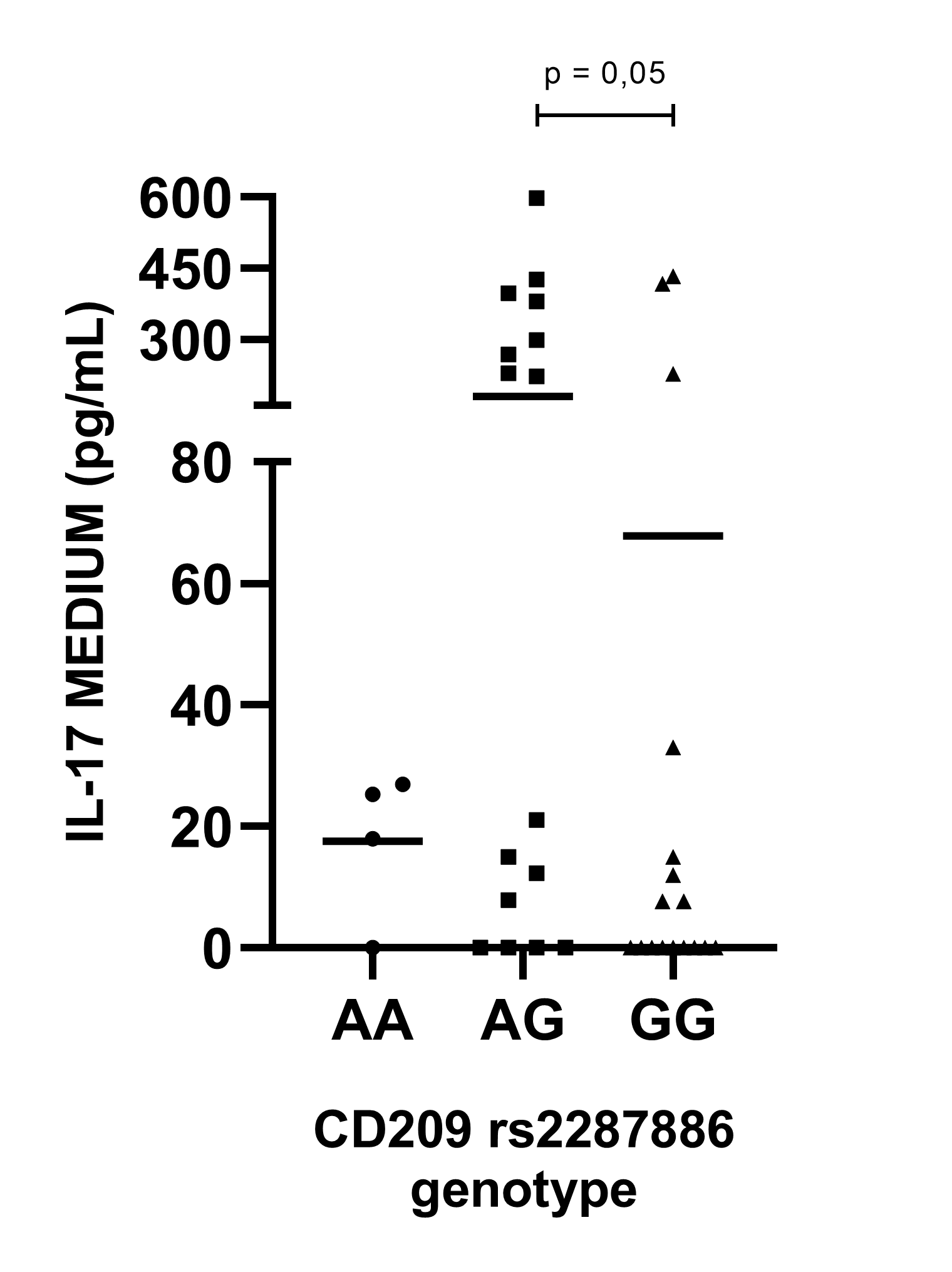

Supplement: Supplementary Figure 1 — CD209 rs2287886 SNP is associated with differences IL-17 in schistosomiasis patients. Levels of IL-17 in 37 schistosomiasis patients stratified by CD209 rs2287886 genotype. *p < 0.05 (Mann-Whitney test). [file Image_1.tif]

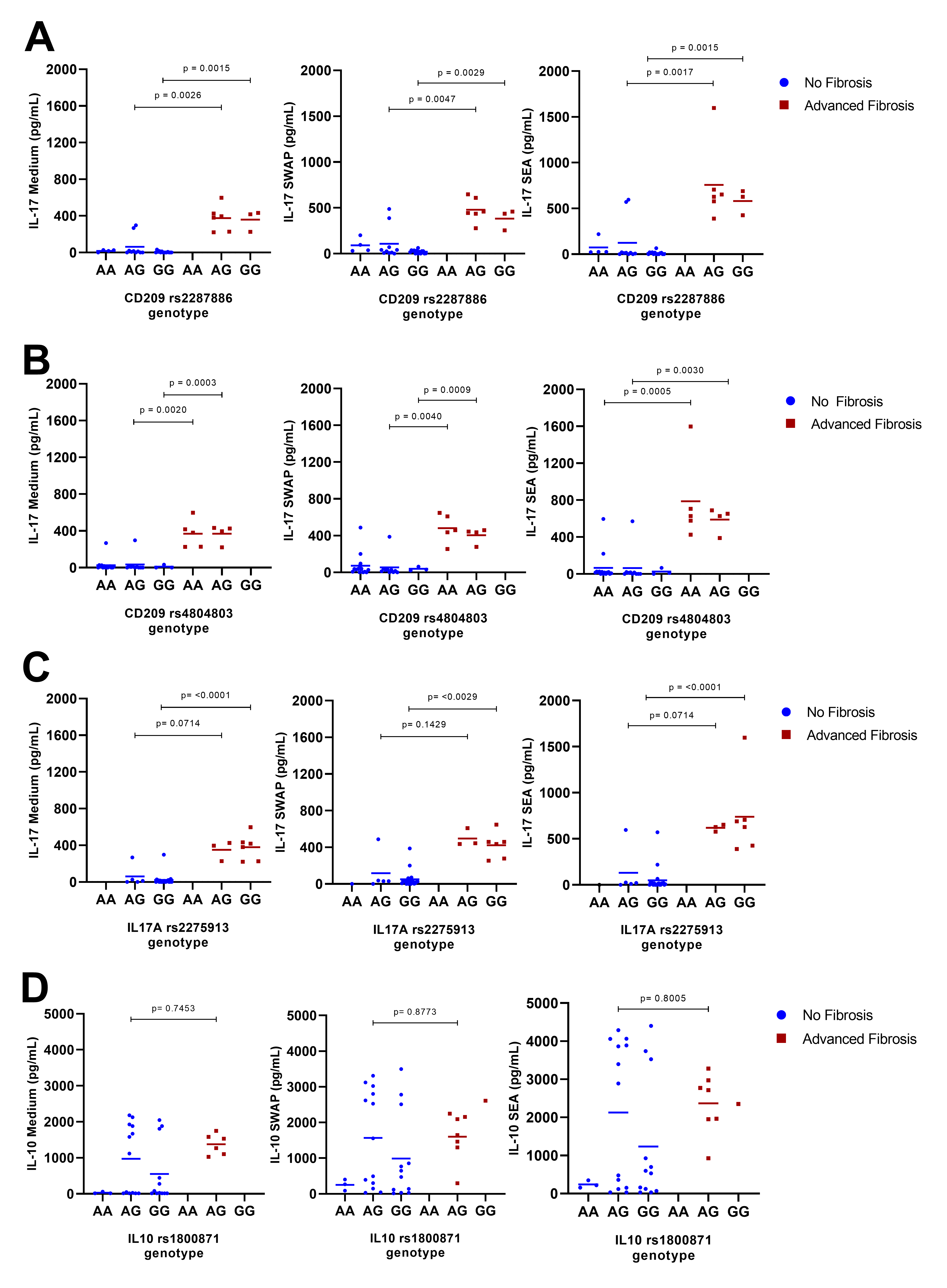

Supplement: Supplementary Figure 2 — Cytokine levels in no fibrosis and advanced fibrosis schistosomiasis patients by different genotypes to IL17A rs2275913, CD209 rs2287886, CD209 rs4804803 and IL10 rs1800871 SNP. Comparisons between the levels of IL-17 and IL-10 cytokines in supernatants from PBMC cultures not stimulated (Medium) or stimulated with adult worm (SWAP) and egg (SEA) S. mansoni antigens from schistosomiasis patient stratified by groups of advanced fibrosis (n=9) and no fibrosis (n=28) and by genotypes to CD209 rs2287886 (A), CD209 rs4804803 (B), IL-17A rs2275913 (C), and IL-10 rs1800871(D). *p < 0.05 (Mann-Whitney test). [file Image_2.tif]
