# Supplementary material for: Association of IL-9, IL-10, and IL-17 Cytokines With Hepatic Fibrosis in Human Schistosoma mansoni Infection
Source: Front Immunol. 2021 Dec 14;12:779534. doi: 10.3389/fimmu.2021.779534 (PMC8712476; doi:10.3389/fimmu.2021.779534)
Supplement: Supplementary file 3 [file Table_1.docx]

| **Supplementary Table 1. Identification and characterization of genotyped single nucleotide polymorphisms (SNPs)** | | | | | |
| --- | --- | --- | --- | --- | --- |
| **Gene** | **SNP** | **Chromosome / Position** | **SNP type** | **Allele** | **SNP** ***Assay*** |
| IL17A | rs2275913 | Chr 6/ Intragenic | Transition substitution | G/A | C__15879983_10 |
| IL10 | rs1800871 | Chr 1/ Intragenic | Transition substitution | A/G | C___1747362_10 |
| IL10 | rs1800872 | Chr 1/ Intragenic | Transversion substitution | T/G | C___1747363_10 |
| CD209 | rs2287886 | Chr 19/ Intragenic | Transition substitution | A/G | C__11515683_1_ |
| CD209 | rs4804803 | Chr 19/ Intragenic | Transition substitution | AG | C___1999340_10 |
