# Supplementary material for: Association of IL-9, IL-10, and IL-17 Cytokines With Hepatic Fibrosis in Human Schistosoma mansoni Infection
Source: Front Immunol. 2021 Dec 14;12:779534. doi: 10.3389/fimmu.2021.779534 (PMC8712476; doi:10.3389/fimmu.2021.779534)
Supplement: Supplementary file 4 [file Table_2.docx]

**Supplementary Table 2. Correlation values (Spearman's R) between ultrasound parameters and serum level of cytokines in patients with schistosomiasis**.

|  | **Average periportal thickening** | **Portal vein diameter** | **Spleen size** | **Left hepatic lobe size** | **Right hepatic lobe size** |
| --- | --- | --- | --- | --- | --- |
| **IL-9 SEA** | R = 0.44  (p = 0.008) | R = 0.47  (p = 0.003) | R = 0.45  (p = 0.005) | R = 0.25  (p = 0.13) | R = -0.05  (p = 0.77) |
| **IL-17 SEA** | R = 0.44  (p = 0.003) | R = 0.49  (p = 0.0008) | R = 0.45  (p = 0.002) | R = 0.01  (p = 0.92) | R = -0.26  (p = 0.08) |
| **IL-17 SWAP** | R = 0.11  (p = 0.41) | R = 0.19  (p = 0.15) | R = 0.11  (p = 0.42) | R = 0.07  (p = 0.59) | R = -0.04  (p = 0.71) |
| **IFN-γ SEA** | R = -0.27  (p = 0.02) | R = 0.02  (p = 0.86) | R = -0.25  (p = 0.03) | R = 0.29  (p = 0.01) | R = 0.22  (p = 0.06) |
| **IFN-γ SWAP** | R = -0.22  (p = 0.07) | R = 0.10  (p = 0.40) | R = -0.09  (p = 0.45) | R = 0.33  (p = 0.005) | R = 0.31  (p = 0.01) |
| **TNF-α SEA** | R = -0.12  (p = 0.34) | R = 0.16  (p = 0.22) | R = -0.07  (p = 0.60) | R = 0.14  (p = 0.29) | R = 0.29  (p = 0.02) |
| **TNF-α SWAP** | R = -0.08  (p = 0.53) | R = 0.15  (p = 0.27) | R = -0.06  (p = 0.66) | R = 0.11  (p = 0.41) | R = 0.16  (p = 0.21) |
| **IL-5 SEA** | R = -0.05  (p = 0.66) | R = 0.16  (p = 0.19) | R = -0.06  (p = 0.58) | R = 0.19  (p = 0.10) | R = 0.19  (p = 0.11) |
| **IL-5 SWAP** | R = -0.004  (p = 0.97) | R = 0.09  (p = 0.43) | R = 0.02  (p = 0.84) | R = 0.18  (p = 0.12) | R = 0.03  (p = 0.77) |
| **IL-10 SEA** | R = 0.30  (p = 0.01) | R = 0.21  (p = 0.08) | R = 0.17  (p = 0.16) | R = -0.07  (p = 0.56) | R = -0.03  (p = 0.77) |
| **IL-10 SWAP** | R = 0.15  (p = 0.23) | R = 0.18  (p = 0.14) | R = 0.16  (p = 0.21) | R = -0.006  (p = 0.95) | R = 0.05  (p = 0.67) |
