# Supplementary material for: Association of IL-9, IL-10, and IL-17 Cytokines With Hepatic Fibrosis in Human Schistosoma mansoni Infection
Source: Front Immunol. 2021 Dec 14;12:779534. doi: 10.3389/fimmu.2021.779534 (PMC8712476; doi:10.3389/fimmu.2021.779534)
Supplement: Supplementary file 5 [file Table_3.docx]

| **Supplementary Table 3. Frequency and distribution of SNPs in advanced fibrosis and no fibrosis patients** | | | | | | | | | | | | |
| --- | --- | --- | --- | --- | --- | --- | --- | --- | --- | --- | --- | --- |
|  | Allele and genotype frequencies (%)^a^ | | | | |  |  | | |  | |  |
|  |  | Advanced Fibrosis | | No fibrosis | |  |  | | |  | |  |
| IL17A  rs2275913  IL10  rs1800871 |  | *n* = 9 | | *n* = 28 | | Allele/Genotype | OR  95% CI | | | | | *p^b^* |
|  | A | 3 (16.7) | | 7 (12.5) | | A vs. G | 1.400 | | | | 0.357- 6.412 | 0.697 |
|  | G | 15 (83.3) | | 49 (87.5) | |  |  |  |  |  |  |  |
|  |  |  | |  | |  |  | |  | | |  |
|  | AA | 0 (0) | | 1 (3.57) | |  |  | | | | |  |
|  | AG | 3 (33.3) | | 5 (17.85) | | AA + AG vs. GG | 1.833 | | | | 0.401 - 10.47 | 0.657 |
|  | GG | 6 (66.7) | | 22 (78.58) | |  |  | | | |  |  |
|  |  |  | |  | |  |  | | | | |  |
|  | A | 8 (44.4) | | 19 (33.9) | | A vs. G | 1.558 | | | | 0.515 - 4.557 | 0.574 |
|  | G | 10 (55.6) | | 37 (66.1) | |  |  |  |  |  |  |  |
|  |  |  | |  | |  |  | |  | | |  |
|  | AA | 0 (0) | | 3 (10.7) | |  |  | | | | |  |
|  | AG | 8 (88.9) | | 13 (46.4) | | AA + AG vs. GG | 6.000 | | | | 0.816 – 71.42 | 0.119 |
|  | GG | 1 (11.1) | | 12 (42.9) | |  |  | | | |  |  |
|  |  | |  | |  |  | |  | | |  |  |
|  |  | |  | |  |  | |  | | |  |  |
|  | T | | 8 (44.4) | | 19 (33.9) | T vs. G | | 1.558 | | | 0.515 - 4.557 | 0.574 |
|  | G | | 10 (55.6) | | 37 (66.1) |  | |  | | |  |  |
| IL10 |  | | | | | | | | | | | |
| rs1800872 | TT | | 0 (0) | | 3 (10.7) |  | |  | | |  |  |
|  | TG | | 8 (88.9) | | 13 (46.4) | TT + TG vs. GG | | 6.000 | | | 0.816 – 71.42 | 0.119 |
|  | GG | | 1 (11.1) | | 12 (42.9) |  | |  | | |  |  |
|  |  | |  | |  |  | |  | | |  |  |
|  | A | | 6 (33.3) | | 18 (34.6) | A vs. G | | 0.944 | | | 0.285 – 2.851 | >0.9999 |
|  | G | | 12 (66.7) | | 34 (65.4) |  | |  | | |  |  |
| CD209 |  | |  | |  |  | |  | | |  |  |
| rs2287886 | AA | | 0 (0) | | 4 (14.3) |  | |  | | |  |  |
|  | AG | | 6 (66.7) | | 10 (35.7) | AA + AG vs. GG | | 2.000 | | | 0.450 - 8.293 | 0.462 |
|  | GG | | 3 (33.3) | | 14 (50) |  | |  | | |  |  |
|  |  | |  | |  |  | |  | | |  |  |
|  |  | |  | |  |  | |  | | |  |  |
|  | G | | 4 (22.2) | | 16(28.6) | G vs. A | | 0.714 | | | 0.229 - 2.449 | 0.763 |
|  | A | | 14 (77.8) | | 40 (71.4) |  | |  | | |  |  |
| CD209 |  | |  | |  |  | |  | | |  |  |
| rs4804803 | GG | | 0 (0) | | 3 (10.7) |  | |  | | |  |  |
|  | AG | | 4 (88.9) | | 10 (35.7) | GG + AG vs. AA | | 0.923 | | | 0.242 - 4.328 | >0.9999 |
|  | AA | | 5 (11.1) | | 15 (53.6) |  | |  | | |  |  |
| Abbreviations: OR, odds ratio; CI, confidence interval; ^a^ (%) percentual of the subjects with the specified allele or genotype. ^b^ Test for association were performed using Fisher exact test. | | | | | | | | | | | | |
